# Using *k*-dependence causal forest to mine the most significant dependency relationships among clinical variables for thyroid disease diagnosis

**DOI:** 10.1371/journal.pone.0182070

**Published:** 2017-08-17

**Authors:** LiMin Wang, FangYuan Cao, ShuangCheng Wang, MingHui Sun, LiYan Dong

**Affiliations:** 1 Key Laboratory of Symbolic Computation and Knowledge Engineering of Ministry of Education, Jilin University, ChangChun City 130012, China; 2 Lixin Accounting Research Institute, Shanghai Lixin University of Commerce, Shanghai City 201620, China; King Abdullah University of Science and Technology, SAUDI ARABIA

## Abstract

Numerous data mining models have been proposed to construct computer-aided medical expert systems. Bayesian network classifiers (BNCs) are more distinct and understandable than other models. To graphically describe the dependency relationships among clinical variables for thyroid disease diagnosis and ensure the rationality of the diagnosis results, the proposed *k*-dependence causal forest (KCF) model generates a series of submodels in the framework of maximum spanning tree (MST) and demonstrates stronger dependence representation. Friedman test on 12 UCI datasets shows that KCF has classification accuracy advantage over the other state-of-the-art BNCs, such as Naive Bayes, tree augmented Naive Bayes, and *k*-dependence Bayesian classifier. Our extensive experimental comparison on 4 medical datasets also proves the feasibility and effectiveness of KCF in terms of sensitivity and specificity.

## Background

Data mining [1] [2] is used to extract unknown but potentially useful information by using available incomplete, noisy, fuzzy, and random practical application data. The medical domain consists of a considerable amount of data, including complete human genetic code information; clinical information on the history of patients, diagnosis, inspection, and treatment; and drug management information. Data mining can be applied in the medical field to analyze medical data, extract implicit valuable information, provide correct diagnosis and treatment, and study the genetic law of human diseases and health [3].

While dealing with a large amount of historical information of patients in the database, data mining needs to confirm the diagnosis based on age, gender, auxiliary examination results, and physiological and biochemical indicators of patients. Thus, data mining should eliminate interference of human factors and establish diagnosis rules with good universality, provided that large amounts of data are analyzed in the process. Consequently, researchers can establish a prediction model, test it, and construct an accurate algorithmic model, which can be used for diagnosis of clinical medical conditions.

Now, about 20 million Americans have some form of thyroid disease, and people of all ages and races can have the chance to get thyroid disease [4]. Recently, a fair mount of data mining methods have been investigated to diagnose this kind of disease. To explore the value of contrast-enhanced ultrasound combined with conventional ultrasound in the diagnosis of thyroid microcarcinoma, multivariate logistic regression analysis is performed to determine independent risk factors [5]. Proper interpretation of the thyroid data besides clinical examination and complementary investigation is an important issue, a comparative study of thyroid disease diagnosis is made by using three different types of neural networks, i.e. multilayer neural network, probabilistic neural network and learning vector quantization neural network [6]. An enhanced fuzzy *k*-nearest neighbor (FKNN) classifier based computer aided diagnostic system is presented for thyroid disease [4]. The neighborhood size *k* and the fuzzy strength parameter *m* in FKNN classifier are adaptively specified by the particle swarm optimization approach. The application of Support Vector Machines is proposed to classify thyroid bioptic specimens [7], together with a particular wrapper feature selection algorithm (i.e., recursive feature elimination). The model is able to provide an accurate discriminatory capability using only 20 out of 144 features, resulting in an increase of the model performances, reliability, and computational efficiency. To elucidate the cytological characteristics and the diagnostic usefulness of intraoperative cytology for papillary thyroid carcinoma, decision tree analysis is used to find effective features for accurate cytological diagnosis [8].

Bayesian method is an intelligent computing method used in reasoning and managing uncertainty problems [9]. BNC is a probability network based on graphical models used to provide probabilistic inference, thus it is more distinct and understandable than other methods. A BNC consists of a structural model and a set of conditional probabilities. The structural model is a directed acyclic graph, in which nodes represent classes *C* and a set of random attributes **X** = (*X*_1_, *X*_2_, …, *X*_*n*_). Arcs between nodes are used to describe the conditional dependence relationships, which are quantified using conditional probabilities for each node given to the parents. Bayesian methods have gained increasing interest in medical diagnosis. BN and graph theory are used to encode causal relations among variables for diagnosis and predictions in the medical domain [10–12].

**Fig 1:**
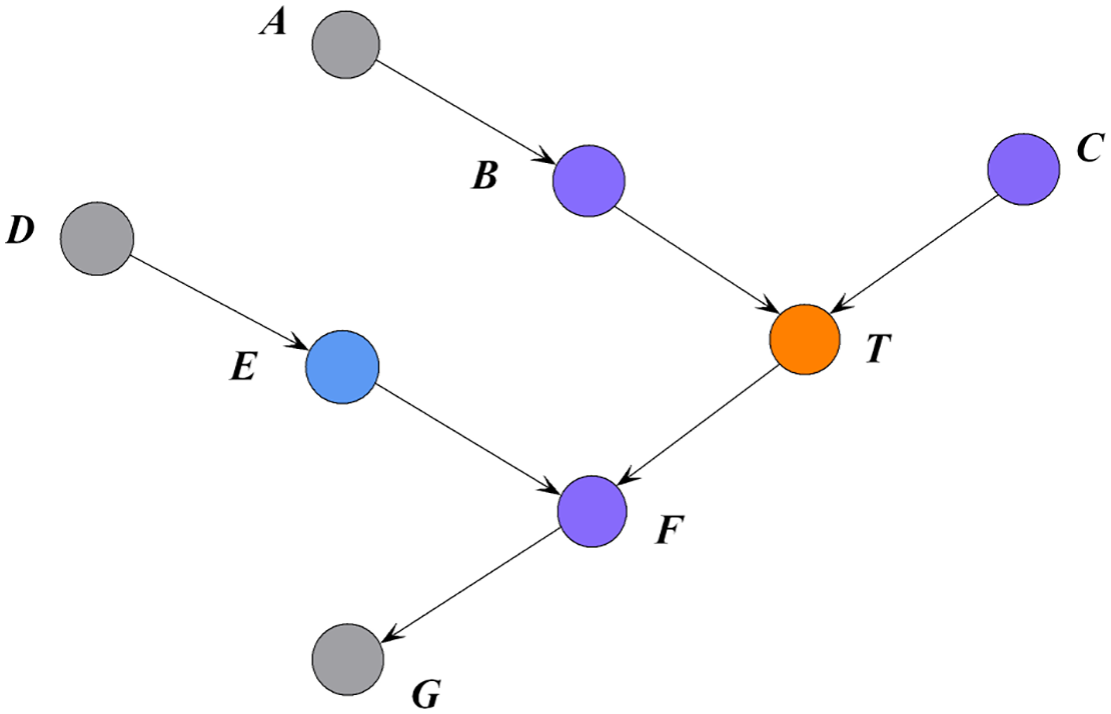
An example Markov blanket.

The Markov blanket of a target attribute is the minimal attribute set for explaining the target attribute based on the conditional independence of all the attributes to be connected in a BN [13]. Koller and Sahami [14] defined the Markov blanket of a target attribute as the minimal set of conditioned attributes, in which all other attributes are independent of the target attribute in the probabilistic graphical model. Hence, the Markov blanket of a target attribute removes unnecessary attributes and represents the minimal information for explaining the target attribute. In a BN model, the Markov blanket of *T*, i.e., MB(*T*) is the union of parent, child, and parent of children nodes of *T* [13, 15]. For example, in Fig 1, the parent nodes of *T* are *B* and *C*, the child node of *T* is *F*, and the parent of the children node of *T* is *E*. Thus, the Markov blanket of *T* is MB(*T*) = {*B*, *C*, *F*, *E*}, indicating that nodes *A*, *D*, and *G* are independent of *T* conditioned on MB(*T*).

The performance of a classifier is evaluated using two key factors, namely, classification accuracy and space complexity of a model. A BN cannot express all relationships between the attributes and the class. Thus, a trade-off should exist between the structure complexity and classification accuracy. Some restricted Bayesian classifiers, e.g., Naive Bayes (NB), tree augmented Naive Bayes (TAN), and *k*-dependence BNs (KDB), exhibit satisfactory performance for classification at different levels of conditional independence assumption. When carrying out medical analysis, different doctors may consider different factor or attribute as starting point. One BNC is unable to express this diversity. This paper proposes a novel learning algorithm called the *k*-dependence causal forest (KCF). This algorithm generates a series of submodels, which are used to construct classifiers with different root nodes at arbitrary points (values of *k*) along the attribute dependence spectrum. The KCF algorithm aims to describe the significant dependency relationships between root node *X*_*r*_ and MB(*X*_*r*_) while simultaneously providing accurate diagnosis to patients with thyroid diseases.

## Materials and methods

### Data

This research work adopts the public thyroid disease dataset from the University of California, Irvine (UCI) Machine Learning Repository [16]. The UCI database currently contains 335 datasets, and the number of sets continuously increases. The thyroid disease dataset was stored in the UCI by Ross Quinlan during his visit in 1987 for the 1987 Machine Learning Workshop; the set contains 9172 real historical instances. Each instance consists of 29 attributes, which can be classified into 20 classes. The characteristics of thyroid disease dataset are multivariate and domain theory, the characteristics of the contained attributes are categorical and real, and the associated task of the dataset is classification.

### Three restricted Bayesian classifiers

BNs are often used to solve classification problems by constructing classifiers from a given set of training instances with class labels. With high classification accuracy and efficiency, BN classifiers perform outstandingly in a number of classification methods. This paper briefly introduces the three popular restricted Bayesian classifiers. In the following discussion, capital letters, such as *X*, *Y* and *Z*, denote attribute names, and lower-case letters, such as *x*, *y* and *z*, denote the specific values taken by those attributes. Sets of attributes are denoted by boldface capital letters, such as X,Y and Z, and assignments of values to the attributes in these sets are denoted by boldface lowercase letters, such as **x, y** and **z**.

The NB classifier is the simplest BN model and is very robust [17]. Given the *n* independent attributes **X** = (*X*_1_, *X*_2_, …, *X*_*n*_) and *m* classes *c*_1_, *c*_2_, …, *c*_*m*_, classification will derive the maximum of *P*(*c*_*i*_|*x*), where 1 ≤ *i* ≤ *m*. Result can be derived from the Bayesian theorem, as Eq (1) shows:

$$\begin{array}{c} P\left(c_{i}|x\right)=\frac{P\left(c_{i}\right)P\left(x|c_{i}\right)}{P(x)} \end{array}$$ (1)

The rigorous assumption in NB is that all attributes are conditionally independent of each other. Thus, the class assignments of the test samples are based on Eq (2).

$$\begin{array}{c} \arg\max_{c_{i}}P\left(c_{i}\right)P\left(x|c_{i}\right)=\arg\max_{c_{i}}P\left(c_{i}\right)\prod_{j=1}^{n}P\left(x_{j}|c_{i}\right) \end{array}$$ (2)

**Fig 2:**
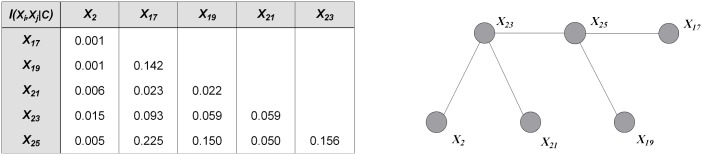
An example of conditional mutual information matrix (a) and corresponding undirected MST (b). Attributes {*X*_2_, *X*_17_, *X*_19_, *X*_21_, *X*_23_, *X*_25_} correspond to clinical variables *on thyroxine*, *TSH*, *T3*, *TT4*, *T4U* and *FTI*, respectively.

The basic framework of TAN [18] is the extension of the Chow-Liu tree [19], which utilizes conditional mutual information to build a maximum spanning tree (MST). TAN is a one-dependence classifier because it allows each attribute to have at most one parent in addition to the class. In practice, TAN is regarded as a good trade-off between the model complexity and classification performance. Fig 2 shows an example of the condition mutual information matrix with six attributes and corresponding undirected MST. The selected six attributes are the first few attributes with the maximum mutual information with class *I*(*X*_*i*_; *C*) in the thyroid disease dataset.

For a TAN model, the class assignments of the test samples are based on Eq (3).

$$\begin{array}{c} \arg\max_{c_{i}}P\left(c_{i}\right)P\left(x|c_{i}\right)=\arg\max_{c_{i}}P\left(c_{i}\right)\prod_{j=1}^{n}P\left(x_{j}|c_{i},x_{jp}\right) \end{array}$$ (3)

where *X*_*jp*_ is the parent node of *X*_*j*_.

**Fig 3:**
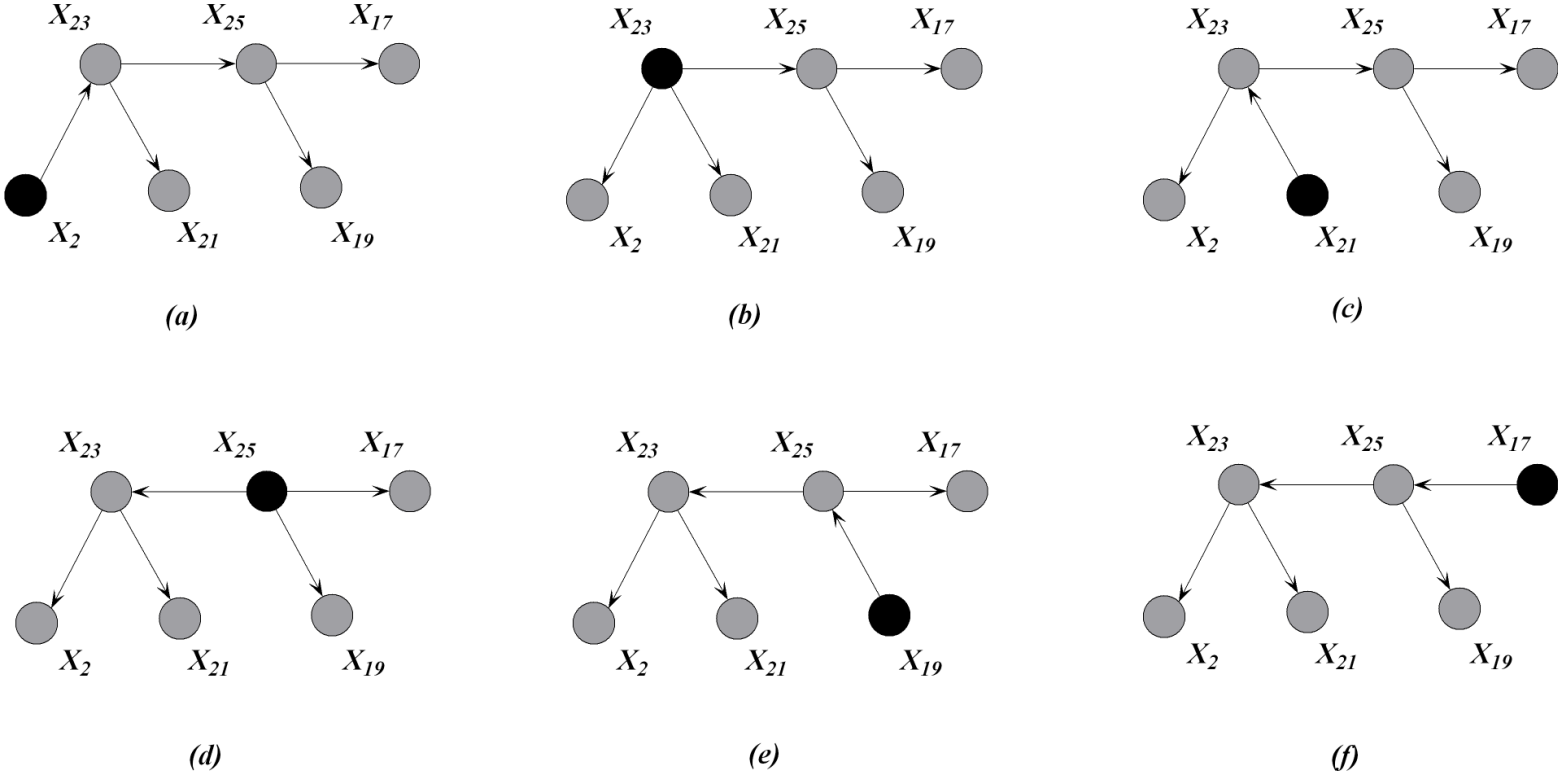
An example of directed MSTs with different root nodes, which are filled in black. Attributes {*X*_2_, *X*_17_, *X*_19_, *X*_21_, *X*_23_, *X*_25_} correspond to clinical variables *on thyroxine*, *TSH*, *T3*, *TT4*, *T4U* and *FTI*, respectively.

After selecting each attribute as the root node and setting the outward direction of all the arcs from the attributes, six different directed MSTs are generated, as shown in Fig 3. The root node is filled in black. The directed MSTs can be regarded as different representations of the same spectrum of causal relationships under different conditions. One MST corresponds to *n* directed trees, and each tree uses different attributes as the root node. Although TAN can achieve a global one-dependence optimization, MST cannot be extended to arbitrary *k*-dependence structure when *k* > 1.

The KDB [20] is a *k*-dependence classifier because it allows each attribute to have a maximum number of *k* parents in addition to the class attribute. Starting with the highest, an attribute order is pre-determined by comparing the mutual information *I*(*X*_*i*_; *C*). By comparing conditional mutual information *I*(*X*_*i*_; *X*_*j*_|*C*), each attribute can select a maximum number of *k* parents among the attributes ahead of itself in the pre-determined order.

For a KDB model, the class assignments of the test samples are based on Eq (4).

$$\begin{array}{c} P\left(X|C_{i}\right)=\prod_{k=1}^{n}P\left(X_{k}|C_{i},X_{i1},\cdots ,X_{ip}\right)\arg\max_{c_{i}}P\left(c_{i}\right)P\left(x|c_{i}\right)=\arg\max_{c_{i}}P\left(c_{i}\right)\prod_{j=1}^{n}P\left(x_{j}|c_{i},x_{j1},\cdots ,x_{jp}\right) \end{array}$$ (4)

where {*X*_*j*1_, ⋯, *X*_*jp*_} are the parent attributes of *X*_*j*_ and *p* = *min*(*j* − 1, *k*).

### KCF algorithm

**Fig 4:**
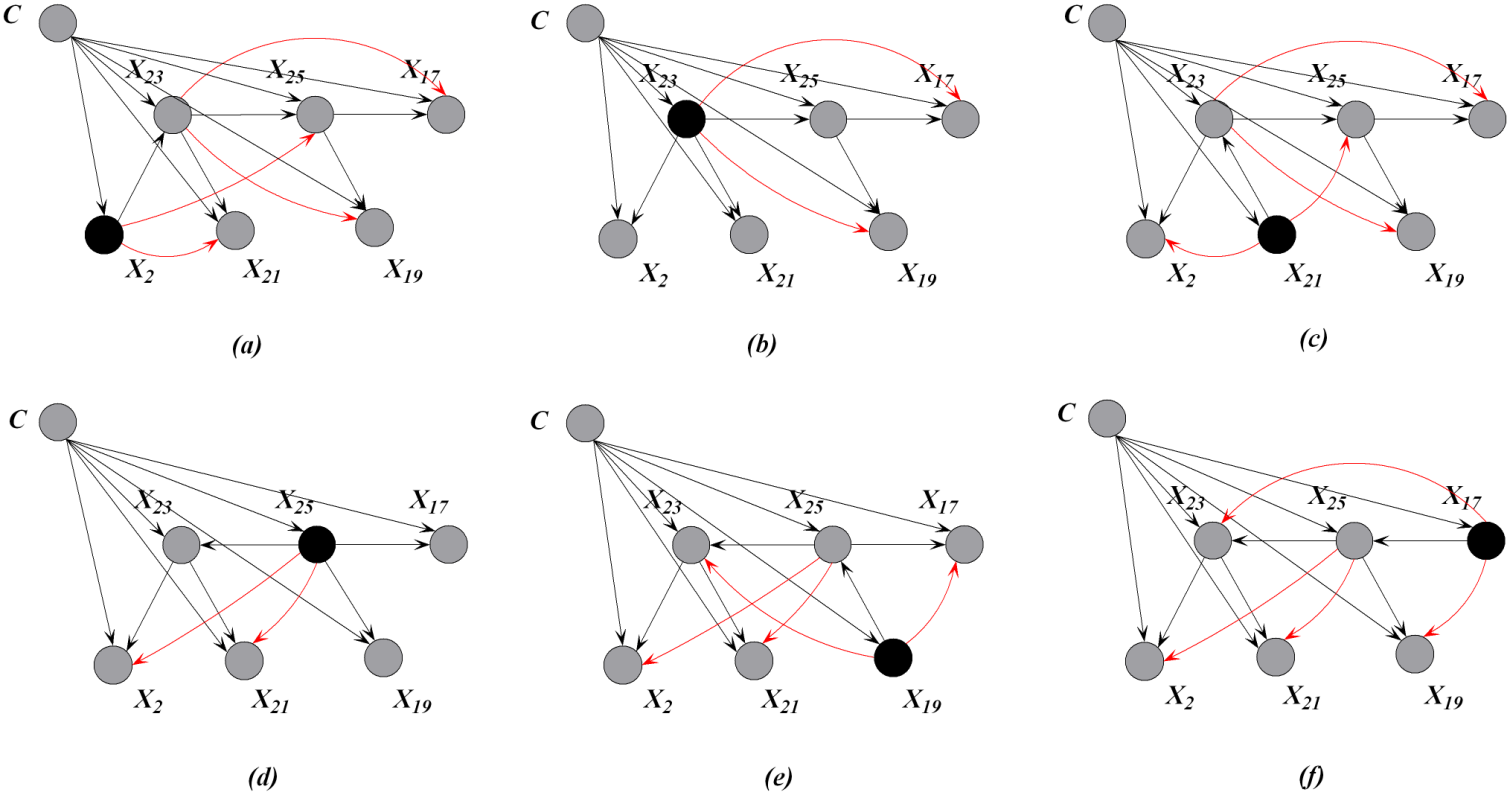
The KCF (*k* = 2) model corresponding to the MSTs shown in Fig 3. Attributes {*X*_2_, *X*_17_, *X*_19_, *X*_21_, *X*_23_, *X*_25_, *C*} correspond to clinical variables *on thyroid*, *TSH*, *T3*, *TT4*, *T4U*, *FTI* and *Class*, respectively.

MST contains the most significant relationships among attributes. Thus at training time, we aim to achieve high-dependence directed trees by extending one-dependence directed trees that are inferred from MST. Each one-dependence directed tree is extended to the *k*-dependence conditional tree along the attribute dependence spectrum. Finally, we will obtain a series of *k*-dependence trees rather than one augmented tree. Leaf node *X*_*i*_ can be used to select other nodes as parents along the path from *X*_*i*_ to the root node by comparing the conditional mutual information. For example, as shown in Fig 3(a), *X*_2_, *X*_23_, *X*_25_ are the possible parents of *X*_17_, and *X*_2_, *X*_23_ are the possible parents of *X*_25_. Different root nodes correspond to different spanning trees or Bayesian classifiers, the ensemble of which finally forms a forest. When *k* > 1, e.g., *k* = 2, more parents can be selected for each non-root node by comparing the conditional mutual information. Fig 4 shows the *k*-dependence Bayesian classifiers when *k* = 2. The newly added arcs are annotated with red color.

At the testing time, KCF estimates the class membership probabilities by using each subclassifier, and the final result is the average of the outputs of all subclassifiers. The training procedure (KCF-Training) and testing procedure (KCF-Testing) are depicted below.

**Algorithm 1** KCF-Training

**Input:** Pre-classified instance set DB with *n* predictive attributes {*X*_1_, ⋯, *X*_*n*_}.

**Output:** Subclassifiers {KCF_1_, ⋯, KCF_*n*_ }.

 1: Compute conditional mutual information *I*(*X*_*i*_; *X*_*j*_|*C*) for each pair of attributes *X*_*i*_ and *X*_*j*_, where *i* ≠ *j*.

 2: Build undirected MST by comparing conditional mutual information.

 3: For each attribute *X*_*i*_(*i* = 1, 2, …, *n*)

  (a) Transform the MST to be a directed one by choosing *X*_*i*_ as the root and setting the direction of all arcs to be outward from it.

  (b) Let the Bayesian subclassifier being constructed, KCF_*i*_, begin with the directed MST.

  (c) Add a node to KCF_*i*_ representing class variable *C*.

  (d) Add an arc from *C* to each node in KCF_*i*_.

  (e) For each node *X*_*j*_(*j* ≠ *i*), add *m* − 1(*m* = *min*(*d*, *k*), *d* is the number of nodes along the branch from root to *X*_*j*_) arcs from *m* − 1 distinct attributes *X*_*P*_ to *X*_*j*_. *X*_*P*_ should locate in the branch from root to *X*_*j*_ and correspond to the first *m* − 1 highest value for *I*(*X*_*P*_; *X*_*j*_|*C*).

 4: Compute the conditional probability tables inferred by the structure of KCF_*i*_ by using counts from *DB*, and output KCF_*i*_.

**Algorithm 2** KCF-Testing

**Input:** KCF_1_, KCF_2_, …, KCF_*n*_ and a testing instance *e*.

**Output:** The conditional probabilities $\hat{P}\left(c|e\right)\left(p=1,2,\ldots ,t\right)$, where *c* is the class label.

 1: For each KCF_*i*(*i* = 1, 2, …, *n*)_, estimate the conditional probability ${\hat{P}}_{i}\left(c|e\right)$ that *e* belongs to class *c*.

 2: Average all of the probabilities $\hat{P}\left(c|e\right)=\frac{1}{n}\sum_{i=1}^{n}{\hat{P}}_{i}\left(c|e\right)$.

 3: Return the estimated $\hat{P}\left(c_{1}|e\right)$, $\hat{P}\left(c_{2}|e\right)$, …, $\hat{P}\left(c_{t}|e\right)$.

*k* is related to the classification performance of a high-dependence classifier. An appropriate value of *k* cannot be effectively preselected to achieve the optimal trade-off between the model complexity and classification performance [21]. For each KCF_*i*_, the space complexity increases exponentially as the value of *k* increases to achieve a trade-off between the classification performance and efficiency. We set *k* = 2 in the following experiments.

## Results

The detailed introduction of the 29 attributes from thyroid disease dataset in UCI database is shown in Table 1. And numeric attributes in thyroid disease dataset are discretized by using 10-bin equal frequency discretization. In order to minimize the bias associated with the random sampling of the training and holdout data samples in comparing the classification accuracy of two or more methods, 10-fold cross-validation is applied to compare the general performance of KCF with three Bayesian network classifiers (i.e., NB, TAN and KDB) and five non-Bayesian network classifiers, i.e., IBK(*k*-Nearest Neighbours) [22], SMO(Support Vector Machine) [23], MultilayerPerception(Artificial Neural Network) [24], DecisionStump(Decision Tree) [25] and SimpleLogistic(linear logistic regression) [26]. In 10-fold cross-validation, whole data are randomly divided to 10 mutually exclusive and approximately equal size subsets. The classification algorithm trained and tested 10 times. In each case, one of the folds is taken as test data and the remaining folds are added to form training data. Thus 10 different test results exist for each training-test configuration. The average of these results gives the test accuracy of the algorithm. All the experiments have been carried out in a C++ software specially designed to deal with out-of-core classification methods. The average classification accuracy (inversely related to zero-one loss [27]) are 75.17%(NB), 80.65%(TAN), 80.43%(KDB), 81.89%(KCF), 78.15%(IBK), 79.67%(SMO), 77.34%(MultilayerPerception), 73.81%(DecisionStump) and 79.53%(SimpleLogistic). Obviously, the proposed KCF algorithm achieves the highest classification accuracy compared with other algorithms and thus performs much more effectively in thyroid disease diagnosis.

**Table 1:** Attributes available for analysis.

Attribute	Type	Explanation	Corresponding symbol in Figs 2–8
age	Numeric	Years	*X*_0_
sex	Binary	Female/male	*X*_1_
on thyroxine	Binary	Yes/no	*X*_2_
query on thyroxine	Binary	Yes/no	*X*_3_
on antithyroid medication	Binary	Yes/no	*X*_4_
sick	Binary	Yes/no	*X*_5_
pregnant	Binary	Yes/no	*X*_6_
thyroid surgery	Binary	Yes/no	*X*_7_
I131 treatment	Binary	Yes/no	*X*_8_
query hypothyroid	Binary	Yes/no	*X*_9_
query hyperthyroid	Binary	Yes/no	*X*_10_
lithium	Binary	Yes/no	*X*_11_
goitre	Binary	Yes/no	*X*_12_
tumor	Binary	Yes/no	*X*_13_
hypopituitary	Binary	Yes/no	*X*_14_
psych	Binary	Yes/no	*X*_15_
TSH measured	Binary	Yes/no	*X*_16_
TSH	Numeric	Thyroid stimulating hormone	*X*_17_
T3 measured	Binary	Yes/no	*X*_18_
T3	Numeric	Triiodothyronine	*X*_19_
TT4 measured	Binary	Yes/no	*X*_20_
TT4	Numeric	Total serum thyroxine	*X*_21_
T4U measured	Binary	Yes/no	*X*_22_
T4U	Numeric	thyroxine	*X*_23_
FTI measured	Binary	Yes/no	*X*_24_
FTI	Numeric	Free Tyroxine Index	*X*_25_
TBG measured	Binary	Yes/no	*X*_26_
TBG	Numeric	Thyroid binding globulin	*X*_27_
referral source	Categorical	WEST, STMW, SVHC, SVI, SVHD, other	*X*_28_
Category	Categorical	20 class labels are divided into 7 groups: Hyperthyroid conditions, Hypothyroid conditions, Binding protein, General health, Replacement therapy, Antithyroid treatment, Miscellaneous	*C*

**Fig 5:**
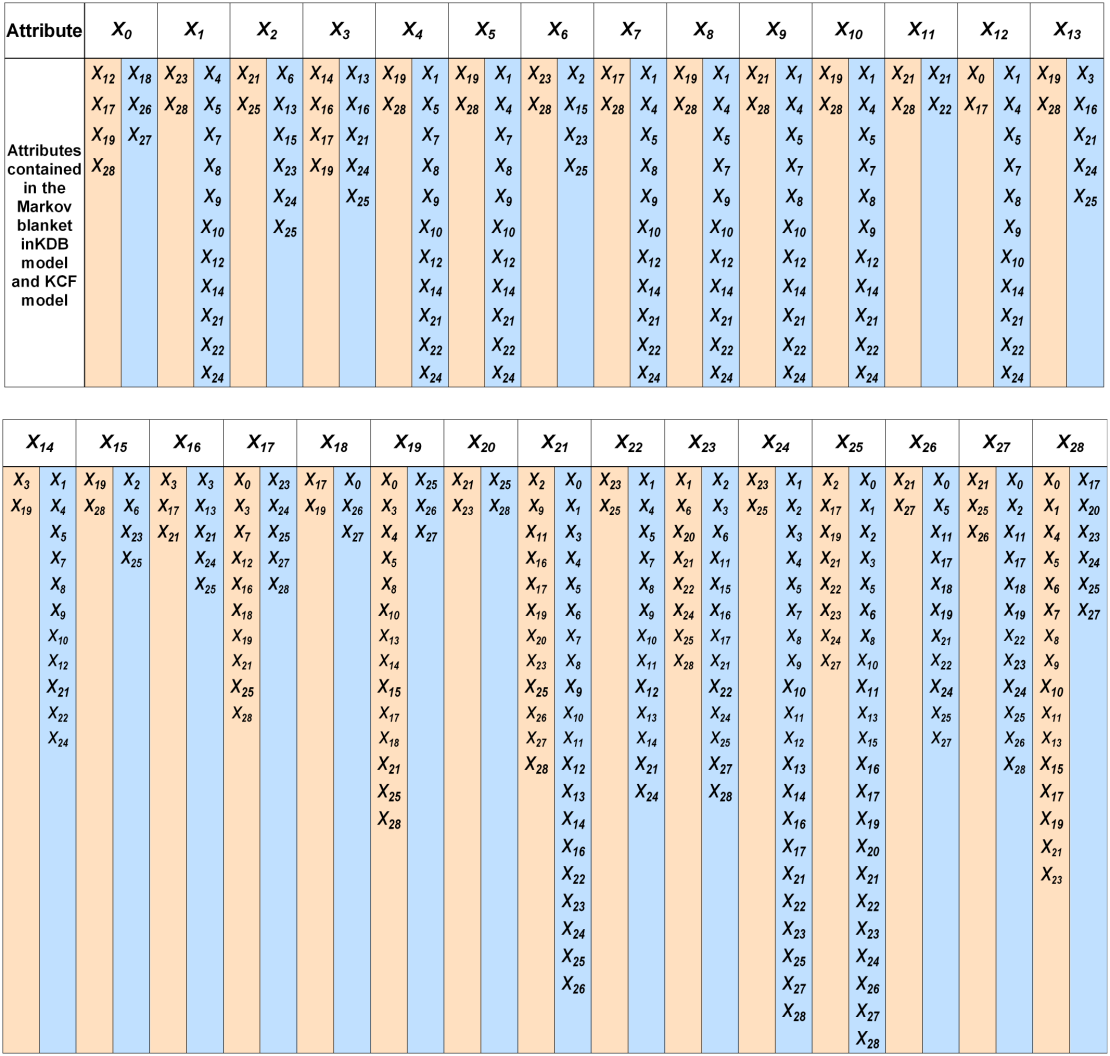
The Markov blanket for KDB (*k* = 2) model is in yellow background and that for KCF (*k* = 2) model is in blue background.

**Fig 6:**
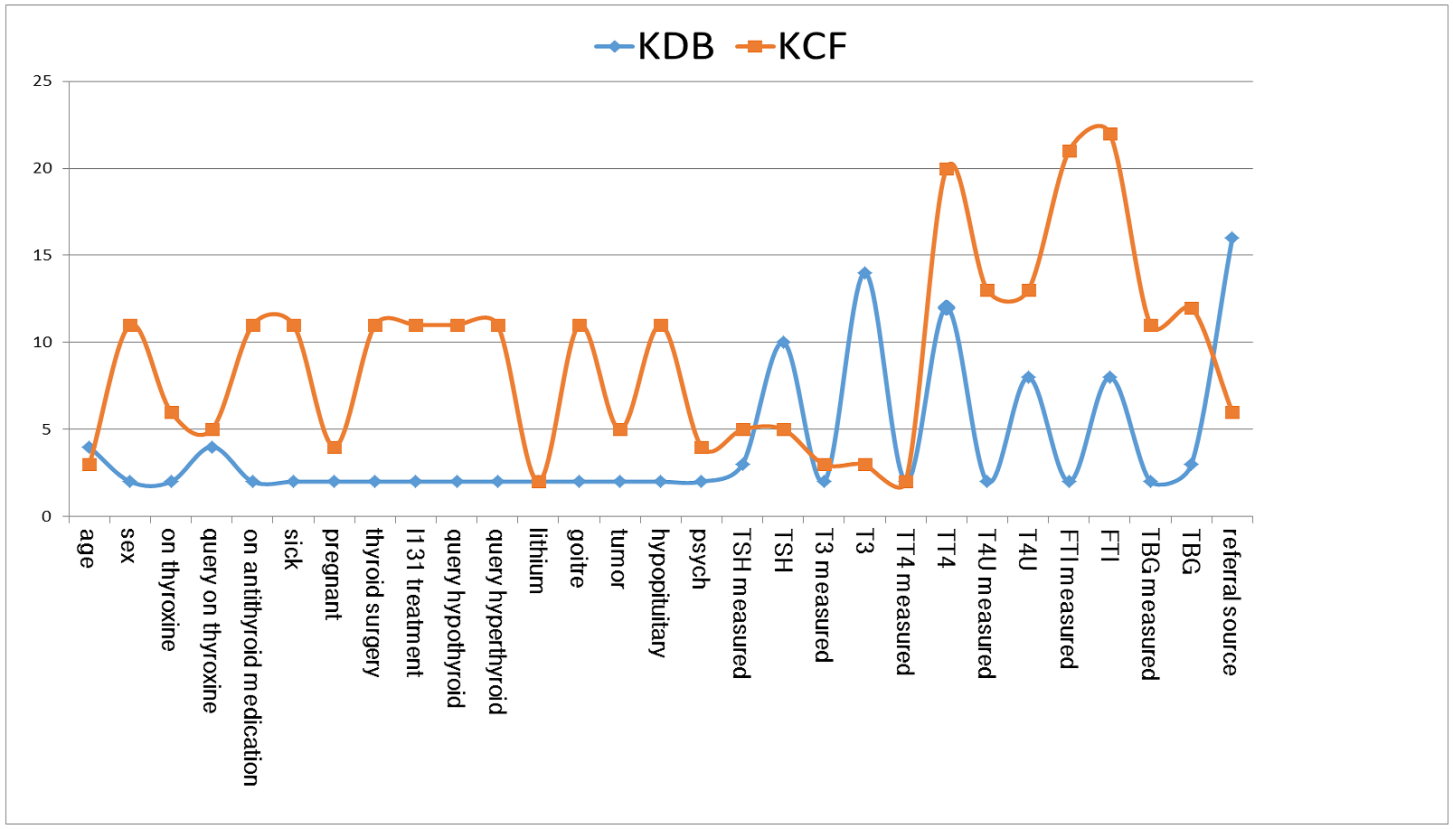
The number of attributes contained in the Markov blanket of each attribute in the KDB (*k* = 2) model and KCF (*k* = 2) model.

To explain the main reason of performance difference of BNCs, we will clarify from the viewpoint of Markov blanket. Compared with low-dependence BNC, high-dependence BNC can demonstrate more conditional dependencies. Thus in the following discussion, we just compare KCF with KDB, both of which are 2-dependence BNCs. KCF will generate a series of submodels, each of which corresponds to different focus for analysis. For example, if *X*_*i*_ is the key factor for diagnosis, then doctors can use the *i*th submodel for further analysis. From the definition of Markov blanket, we can get the following conclusion that *X*_*i*_ is directly and mutually dependent on attributes {*Pa*(*X*_*i*_), *Ch*(*X*_*i*_)} while indirectly dependent on attributes *PC*(*X*_*i*_). The other attributes are useless for further consideration. The time cost for unnecessary analysis and expenditure on unnecessary physical examination will be decreased greatly. With limited time and space complexity, more Markov blanket attributes means more possible dependency relationships to be mined. The list and number of Markov blanket attributes of each attribute for KCF and KDB are shown in Fig 5 and Fig 6, respectively. From Fig 6, for 25 of all of the 29 attributes the number of corresponding Markov blanket attributes for KCF is greater than that for KDB. On average each predictive attribute has 9.1 Markov blanket attributes for KCF, whereas only 4.1 Markov blanket attributes for KDB.

Conditional mutual information *I*(*X*_*i*_; *X*_*j*_|*C*) can be used to quantitatively evaluate the conditional dependence between *X*_*i*_ and *X*_*j*_ given *C*. For any given target attribute *X*_*k*_, *X*_*k*_ is directly dependent on *Pa*(*X*_*k*_) and *Ch*(*X*_*k*_) is directly dependent on *X*_*k*_. Thus the conditional dependencies are measured by *I*(*X*_*i*_; *X*_*k*_|*C*) and *I*(*X*_*j*_; *X*_*k*_|*C*) (*X*_*i*_ ∈ *Pa*(*X*_*k*_), *X*_*j*_ ∈ *Ch*(*X*_*k*_)), respectively. *PC*(*X*_*k*_) is conditionally dependent on *X*_*k*_ but directly dependent on *Ch*(*X*_*k*_). The conditional dependence is measured by $I(X_{i}^{{}^{\prime }};X_{j}^{{}^{\prime }}|C)$ $\left(X_{i}^{{}^{\prime }}\in PC\left(X_{k}\right),X_{j}^{{}^{\prime }}\in Ch\left(X_{k}\right)\right)$. All the conditional dependencies among attributes in *MB*(*X*_*k*_) can then be measured by *MB*_*Info*(*X*_*k*_), which is defined by Eq (5),

$$\begin{array}{ccc} MB\_Info(X_{k}) & = & \sum_{X_{i}\in Pa\left(X_{k}\right)}I\left(X_{i};X_{k}|C\right)+\sum_{X_{j}\in Ch\left(X_{k}\right)}I\left(X_{j};X_{k}|C\right)\\ & & +\sum_{X_{i}^{{}^{\prime }}\in PC\left(X_{k}\right)}\sum_{X_{j}^{{}^{\prime }}\in Ch\left(X_{k}\right)}I\left(X_{i}^{{}^{\prime }};X_{j}^{{}^{\prime }}|C\right) \end{array}$$ (5)

We also compare the average weight of conditional dependencies implicated in *MB*(*X*_*k*_), which is defined by Eq (6),

$$\begin{array}{c} Avg\_MB\_Info\left(X_{k}\right)=\frac{MB\_Info(X_{k})}{number\;of\;attributes\;in\;MB(X_{k})} \end{array}$$ (6)

**Fig 7:**
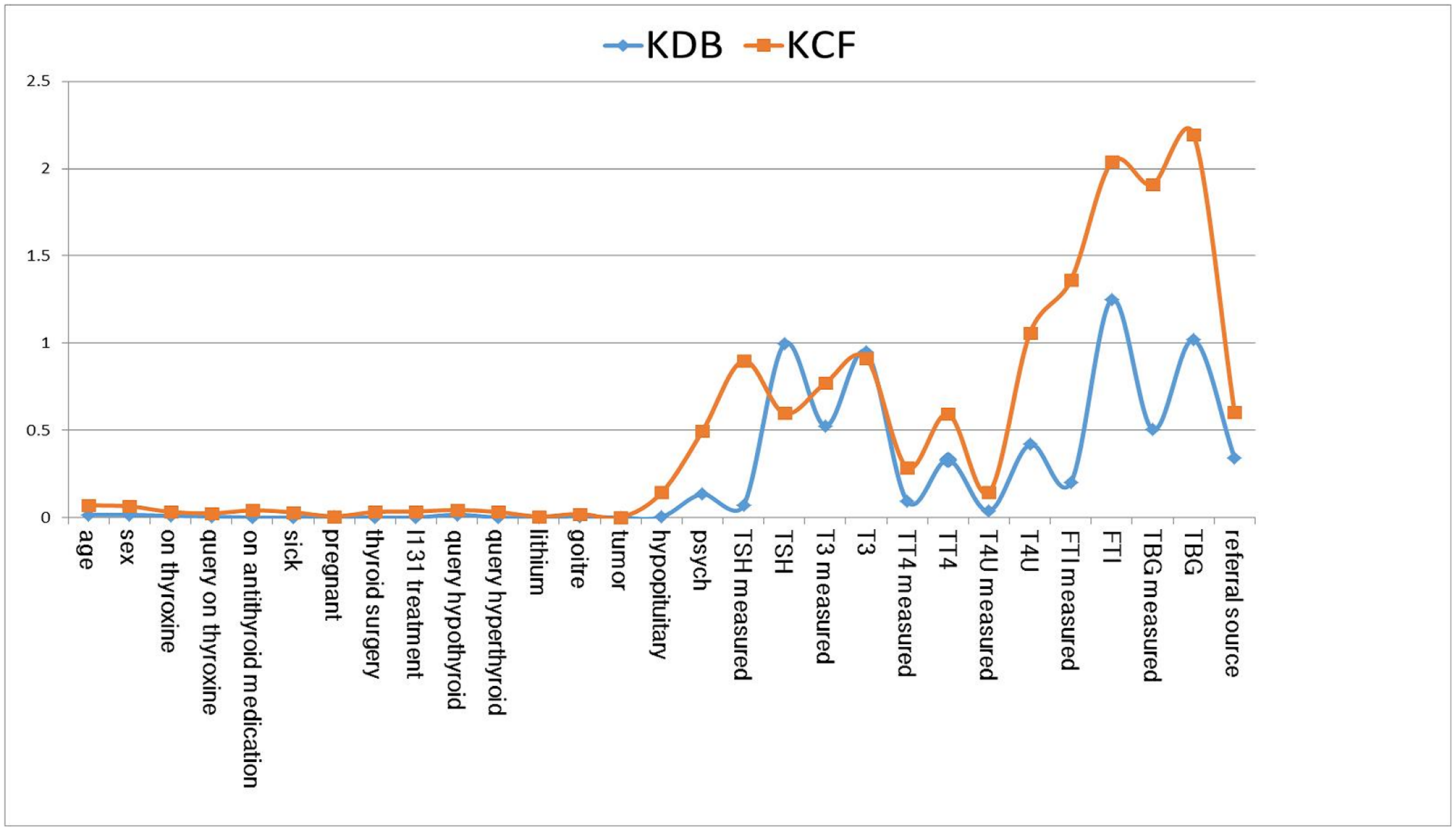
The sum of conditional mutual information between each attribute and the attributes contained in its Markov blanket is shown in (a). The average of conditional mutual information between each attribute and the attributes contained in its Markov blanket is shown in (b).

The comparison results of *MB*_*Info*(*X*_*k*_) between KCF and KDB are shown in Fig 7. For the first 14 attributes, *MB*_*Info*(*X*_*k*_)≈0 {0 ≤ *k* ≤ 13} for both KDB and KCF. Thus *X*_*k*_ {0 ≤ *k* ≤ 13} is directly dependent on class variable whereas independent of any other attributes. For 13 of the other 15 attributes, the value of *MB*_*Info*(*X*_*k*_) {14 ≤ *k* ≤ 28} for KCF is greater than that for KDB. The experimental results prove that KCF can fully demonstrate dependency relationships and thus help to increase the classification accuracy.

## Discussion

Thyroid cancer incidence has been rising since 1978, and its prevalence has increased dramatically over the past decade; currently, thyroid cancer is the fifth most common cancer diagnosed among women. By contrast, the incidence of other malignancies, including lung, colorectal, and breast cancer, decreases [28]. A statistical survey in 2014 showed that 10 million Chinese patients have hyperthyroidism, 90 million have hypothyroidism, more than 100 million are afflicted with thyroid nodules or thyroid cancer, and conservatively; more than 200 million are estimated to have thyroid disease. As the second major disease of the endocrine system, the awareness rate and treatment rate of thyroid diseases are very low in China.

Thyroid nodule is a common clinical problem, and the prevalence of differentiated thyroid cancer increases [29]. Early detection, diagnosis, and treatment are important in curbing the development of thyroid diseases and reducing the mortality rate. Predicting the outcome of diseases and dependency among clinical variables or attributes plays pivotal roles in medical diagnosis and treatment.

For the detailed analysis, this paper calculates and compares the mutual information *I*(*X*_*i*_; *C*) first. The results are sorted starting from the highest. The attribute order is *X*_17_, *X*_25_, *X*_21_, *X*_19_, *X*_23_, *X*_2_, *X*_28_, *X*_27_, *X*_16_, *X*_20_, *X*_26_, *X*_18_, *X*_22_, *X*_24_, *X*_0_, *X*_1_, *X*_6_, *X*_10_, *X*_13_, *X*_7_, *X*_9_, *X*_15_, *X*_4_, *X*_8_, *X*_5_, *X*_3_, *X*_12_, *X*_11_, *X*_14_. From the perspective of medical diagnosis, the attribute with the most intimate relationship with the outcome can be considered as the key attribute and should be the focus of the analysis. The attribute *X*_17_ represents the clinical index for thyroid stimulating hormone (TSH) and should be analyzed initially. TSH can promote the growth of thyroid secreted by adenohypophysis. In addition, TSH can completely improve the function of the thyroid, promoting early release of thyroid hormones and synthesis of T4 and T3.

**Fig 8:**
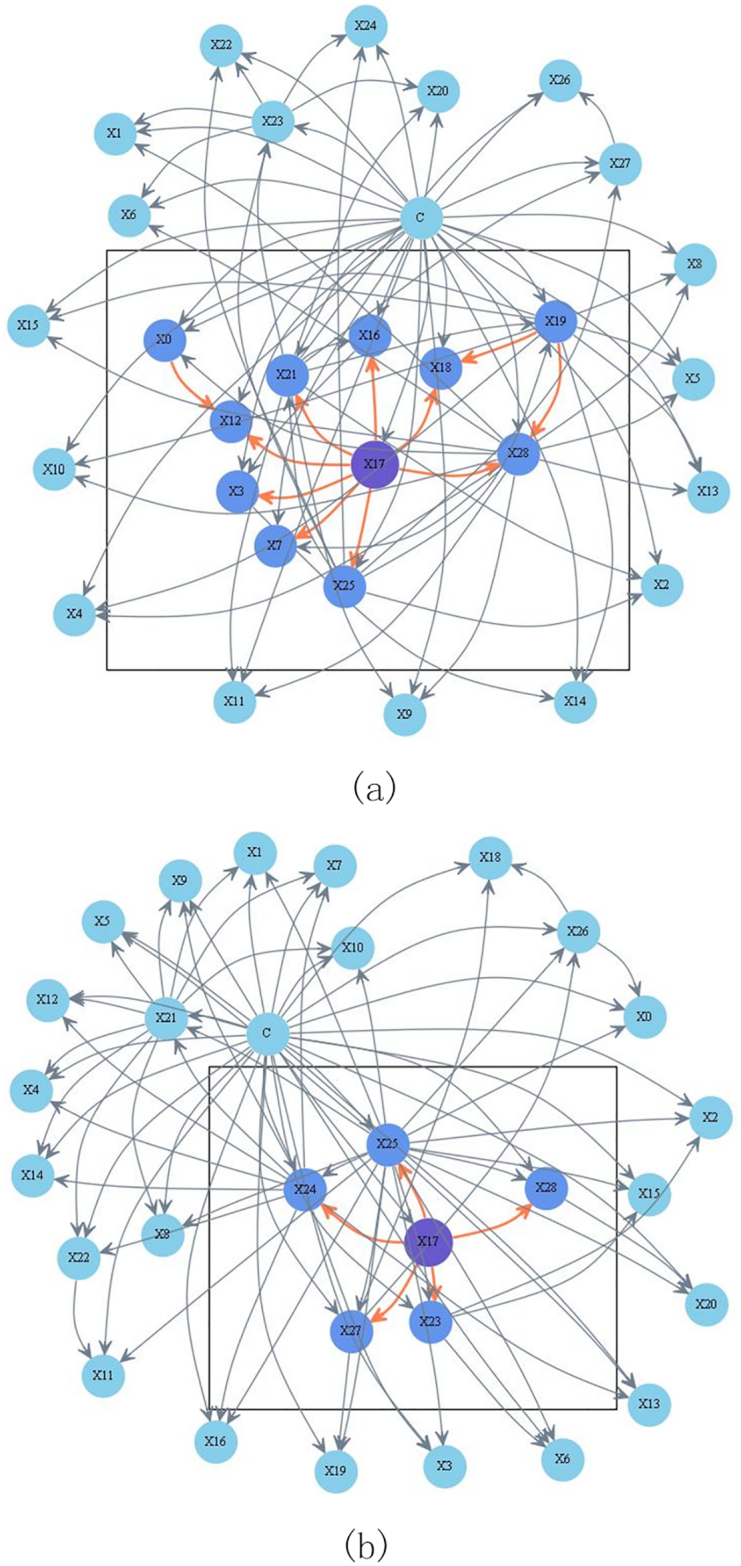
KDB (*k* = 2) model and a submodel of the KCF (*k* = 2) on thyroid disease date set shown respectively in (a) and (b). Attributes {*X*_0_, *X*_1_, ⋯, *X*_28_, *C*} correspond to clinical variables *age*, *sex*, *on thyroxine*, *query on thyroxine*, *on antithyroid medication*, *sick*, *pregnant*, *thyroid surgery*, *I131 treatment*, *query hypothyroid*, *query hyperthyroid*, *lithium*, *goitre*, *tumor*, *hypopituitary*, *psych*, *TSH measured*, *TSH*, *T3 measured*, *T3*, *TT4 measured*, *TT4*, *T4U measured*, *T4U*, *FTI measured*, *FTI*, *TBG measured*, *TBG*, *referral source* and *Class* respectively.

To clarify the role of the TSH attribute, this paper displays the structure of the KDB and a KCF submodel in Fig 8(a) and 8(b), respectively. To make typical and fair comparison, we set *X*_17_ as the common root node of both models. As shown in Fig 8(a), *X*_17_ is the common parent of *X*_25_, *X*_21_, *X*_28_, *X*_16_, *X*_18_, *X*_17_, *X*_3_, and *X*_12_; *X*_0_ and *X*_19_ are the parent nodes of the children of *X*_17_. *X*_0_ is the parent node of *X*_12_, and *X*_19_ is the common parent of *X*_18_ and *X*_28_. *MB*(*X*_17_) contains 10 attributes. *MB*_*Info*(*X*_17_) is 0.902 and *Avg*_*MB*_*Info*(*X*_17_) = 0.09. In the corresponding KCF model shown in Fig 8(b), *X*_17_ is the common parent of *X*_23_, *X*_24_, *X*_25_, *X*_27_, and *X*_28_, whereas *X*_17_ has no parent nodes and no parent of children nodes. Thus, *MB*(*X*_17_) only contains 5 attributes. *MB*_*Info*(*X*_17_) and *Avg*_*MB*_*Info*(*X*_17_) turn to be 0.597 and 0.12, respectively. Similarly, the sum of *MB*_*Info*(*X*_*i*_), i.e., $\sum_{i=0}^{28}MB\_Info\left(X_{i}\right)$, is 14.458 for KCF, whereas it is only 6.964 for KDB. The sum of *Avg*_*MB*_*Info*(*X*_*i*_), i.e., $\sum_{i=0}^{28}Avg\_MB\_Info\left(X_{i}\right)$, is 1.576 for KDB and 1.946 for KCF. Hence, the proposed KCF model describes significant relationships among attributes.

MST contains the most significant dependency relationships, whereas the KDB model can only contain portions of the MST. Additionally, the KCF algorithm can generate a series of submodels rather than one model alone. Thus, for medical diagnosis, any clinical variable or attribute related to thyroid diseases can be regarded as the original cause, and an in-depth research can be conducted on the disease. Hence, the proposed KCF model can handle various patient conditions and is more suitable for providing appropriate treatment compared with a model with a rigid root node generated by other algorithms.

Sensitivity and specificity are statistical measures of the performance of a binary classification test, also known in statistics as classification function. In the context of medical tests sensitivity is the extent to which true positives are not missed/overlooked and specificity is the extent to which positives really represent the condition of interest and not some other condition being mistaken for it. So we select 12 datasets with binary class labels from UCI for comparison of classification accuracy. Table 2 summarizes the characteristics of each dataset, including the numbers of instances, attributes and classes. Averaged One-dependence Estimators (AODE) [30], which utilizes a restricted class of one-dependence estimators and aggregates the predictions of all qualified estimators within this class, is introduced to compare the bagging performance of KCF.

**Table 2:** Datasets.

No.	dataset	Instance	Attribute	Class
1	Echocardiogram	131	6	2
2	Heart*	270	13	2
3	Heart Disease*	303	13	2
4	Chess	551	39	2
5	Breast-cancer-w*	699	9	2
6	Pima-ind-diabetes*	768	8	2
7	Tic-tac-toe	958	9	2
8	German	1000	20	2
9	Spambase	4601	57	2
10	Mushroom	8124	22	2
11	Adult	48842	14	2
12	Census-income	299285	41	2

the datasets denoted with symbol “*” will be used for comparing sensitivity and specificity.

Experimental results of average classification accuracy for different BNCs are shown in Table 3. Friedman test [31], which is a non-parametric measure to compare the ranks of the algorithms for each dataset separately. The ranks of algorithms for each dataset are calculated separately (average ranks are assigned if tied values exist). The null-hypothesis is that all the algorithms performs almost equivalently and there is no significant difference in terms of ranks. The Friedman statistic can be computed as Eq (7) shows,

$$\begin{array}{c} F_{r}=\frac{12}{Nt(t+1)}\sum_{j=1}^{t}R_{j}^{2}-3N\left(t+1\right) \end{array}$$ (7)

where $R_{j}=\sum_{i}r_{i}^{j}$ and $r_{i}^{j}$ is the rank of the *j*-th of *t* algorithms on the *i*-th of *N* datasets. Thus, for any pre-determined level of significance *α* the null hypothesis will be rejected if $F_{r}>\chi_{\alpha }^{2}$, which is the upper-tail critical value having *t* − 1 degrees of freedom. The critical value of $\chi_{\alpha }^{2}$ for *α* = 0.02 is 11.668. With 5 algorithms and 12 datasets, the friedman statistic *F*_*r*_ = 18.55 and *P* < 0.001. Hence the null-hypotheses is rejected again. The average ranks of different classifiers are {NB(1.54), TAN(3.00), AODE(2.54), KDB(3.88), KCF(4.04)}. Thus KCF with the highest rank is the most effective BNC from the perspectives of classification accuracy.

**Table 3:** Experimental results of average classification accuracy for datasets with binary class labels.

Dataset	NB	TAN	KCF	KDB	AODE
Adult	84.2%	86.2%	85.1%	86.2%	86.8%
Breast-cancer-w	95.8%	96.4%	97.4%	95.3%	94.6%
Census-income	76.3%	93.6%	89.9%	94.9%	94.9%
Chess	88.7%	90.7%	90.0%	90.0%	92.4%
Echocardiogram	66.4%	67.2%	67.9%	65.6%	66.4%
German	74.7%	72.7%	75.2%	71.1%	73.0%
Heart	80.2%	80.7%	80.8%	81.9%	80.4%
Heart Disease	79.9%	79.2%	78.8%	77.6%	79.6%
Mushrooms	98.0%	100.0%	100.0%	100.0%	100.0%
Pima-ind-diabetes	75.5%	76.2%	76.2%	75.5%	76.3%
Spambase	89.8%	93.3%	93.3%	93.6%	94.1%
Tic-tac-toe	69.3%	77.1%	73.5%	79.6%	80.6%

When dealing with imbalanced class distribution, traditional classifiers are easily overwhelmed by instances from majority classes while the instances from minority classes are usually ignored. An useful performance measure is the balanced accuracy (BAC) [32] which avoids inflated performance estimates and defined as Eq (8) shows. It is defined as the arithmetic mean of sensitivity and specificity, which are calculated by knowing the *m* binary outputs of the classifiers (indicating membership to given classes). Overall performance is calculated by conducting a leave-one-out test for all training samples.

$$\begin{array}{c} BAC=\frac{sensitivity+specificity}{2} \end{array}$$ (8)

The experimental results of sensitivity, specificity and BAC for BNCs are shown in Table 4. By comparing via two-tailed binomial sign test with a 95% confidence level, Table 5 shows corresponding win/draw/loss (W/D/L) records summarizing the relative BAC of the different BNCs. The W/D/L record in cell [*i*, *j*] of each table contains the number of datasets in which BNC on row *i* has lower, equal or higher outcome relative to the BNC on column *j*. We could see from Table 5 that the bagging mechanism helps AODE increase BAC significantly often relative to TAN and NB. KDB can achieve not only higher classification accuracy but also higher BAC than TAN. KCF utilizes the bagging mechanism of AODE and can represent high-dependence relationships. This may be the main reason why KCF achieves higher BAC more often than the other four BNCs.

**Table 4:** Experimental results of sensitivity, specificity and BAC for medical datasets with binary class labels.

	Dataset	NB	TAN	AODE	KDB	KCF
sensitivity	Breast-cancer-w	0.969	0.973	0.965	0.958	0.971
	Heart	0.840	0.853	0.860	0.853	0.806
	Heart-disease-c	0.829	0.856	0.842	0.816	0.786
	Pima-ind-diabetes	0.820	0.842	0.824	0.838	0.816
specificity	Breast-cancer-w	0.917	0.929	0.945	0.975	0.975
	Heart	0.742	0.750	0.756	0.792	0.854
	Heart-disease-c	0.748	0.741	0.813	0.776	0.846
	Pima-ind-diabetes	0.634	0.612	0.631	0.619	0.642
BAC	Breast-cancer-w	0.943	0.952	0.955	0.966	0.973
	Heart	0.798	0.802	0.808	0.826	0.830
	Heart-disease-c	0.788	0.798	0.802	0.797	0.816
	Pima-ind-diabetes	0.727	0.726	0.727	0.728	0.729

**Table 5:** Win-draw-loss records for different BNCs in terms of BAC.

	Dataset	NB	TAN	AODE	KDB
BAC	TAN	1/3/0		-	-
	AODE	2/2/0	1/3/0	-	-
	KDB	2/2/0	2/2/0	2/1/1	-
	KCF	3/1/0	3/1/0	3/1/0	2/2/0

## Conclusion

Bayesian network can graphically describe the conditional dependencies implicit in training data and Bayesian network classifiers have been previously demonstrated to perform efficiently in medical diagnosis and treatment. One single data mining model cannot deal with all difficult and complicated cases. KCF, which uses the same learning strategy as that of KDB, simultaneously provides *n* submodels rather than one. This improvement helps KCF to describe more significant conditional dependencies. The experimental study on UCI datasets shows that KCF enjoys obvious advantage in classification over other BNCs.
